# Data on primary hydration characteristics of aqueous electrolytes

**DOI:** 10.1016/j.dib.2018.05.037

**Published:** 2018-05-22

**Authors:** Jyoti Sahu, Vinay A. Juvekar

**Affiliations:** Department of Chemical Engineering, Indian Institute of Technology Bombay, Powai, Mumbai 400076, India

## Abstract

The data presented in this article support the research article entitled "Development of a rationale for decoupling osmotic coefficient of electrolytes into electrostatic and nonelectrostatic contributions" (Sahu and Juvekar, 2018) [1]. In this article, we have presented the plots of osmotic coefficients against molality for more than hundred aqueous single electrolytes at 25 °C. The linear regions in these plots are marked to show that they are present in all these electrolytes and that these regions extend over a wide range of concentrations. Slopes of the linear regions are used to estimate the primary molar hydration volume as well as the primary hydration number of these electrolytes. These values are also listed and the method of estimation is presented with sample calculation. These data, not only reinforce the observations made in the main article but also provide useful measures for estimation of the nonelectrostatic contribution to the osmotic coefficient.

**Specifications Table**

**Table:** 

Subject area	*Electrochemistry.*
More specific subject area	*Thermodynamics of electrolytes.*
Type of data	*Plots and Tables*
How data was acquired	*From the analysis of data obtained from published literature*
Data format	*Analyzed*
Experimental factors	*Not Applicable*
Experimental features	*Not Applicable*
Data source location	*Not Applicable*
Data accessibility	*Data are available in this article*
Related research article	*J. Sahu, V.A. Juvekar, Development of a rationale for decoupling osmotic coefficient of electrolytes into electrostatic and nonelectrostatic contributions. Fluid Phase Equilibria 2018, 460: 57-68.*

**Value of the data**

• The linear regions in the osmotic coefficient-molality plots of several aqueous solutions of single electrolytes have been marked. The existence of these linear regions provides supportive evidence to the analysis presented in the main paper [1].
• The primary molar hydration volumes and primary hydration numbers obtained in this article would be useful for estimation of nonelectrostatic contribution to the osmotic coefficient of aqueous solutions of single and mixed electrolytes using the procedure described in Ref. [1].
• These data would also allow estimation of electrostatic contribution to the osmotic coefficient of aqueous solutions of electrolytes using the procedure described in Ref. [1].

## 1 Data

The data are provided in two parts. Part-1 contains plots of osmotic coefficient versus molarity of solutions of single electrolytes. The linear regions are marked on the plots. Slopes of these linear regions are listed below the plots. Part-2 lists the data of the primary molar hydration volume and primary hydration numbers.

Part-1: Plots of Osmotic coefficient-molality data for single aqueous electrolytes.

a. 1-1 electrolytes (data of osmotic coefficient from Ref. [2]).

**Figure:**
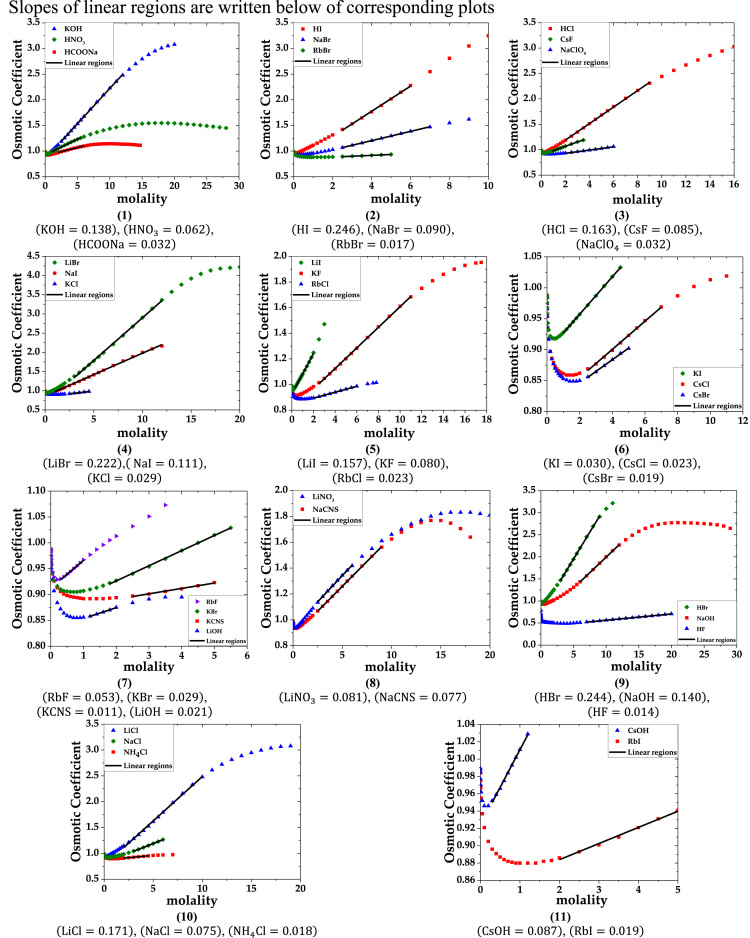

b. 1-2, 2-1, 2-2, 1-3, 3-1 and 2-3 electrolytes (data of osmotic coefficient from Ref. [3]).

**Figure:**
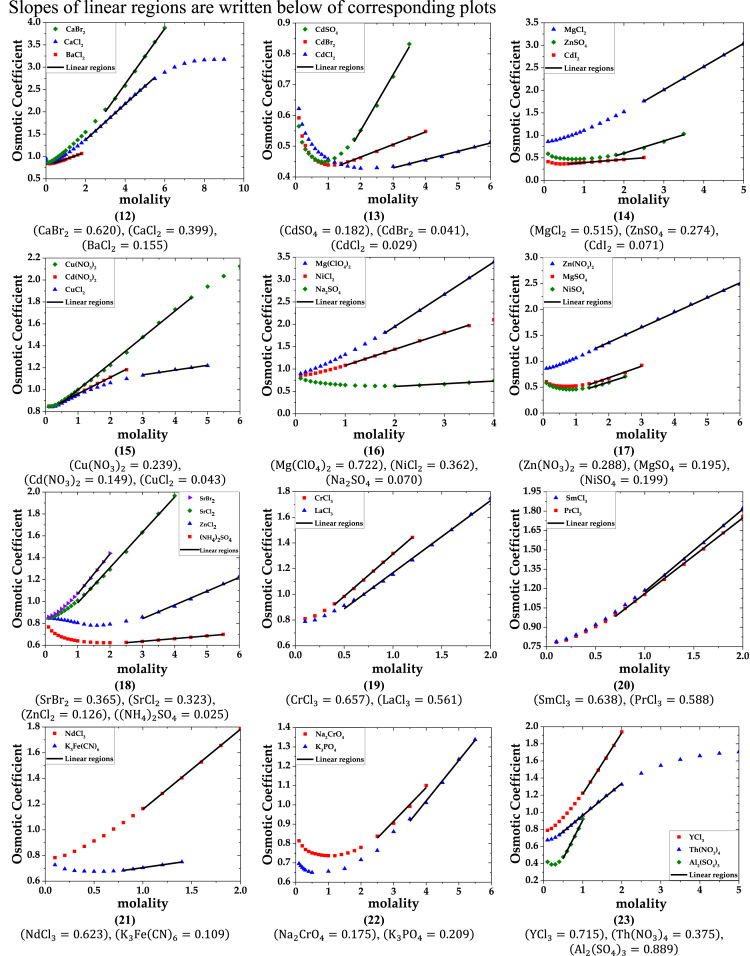

**Figure:**
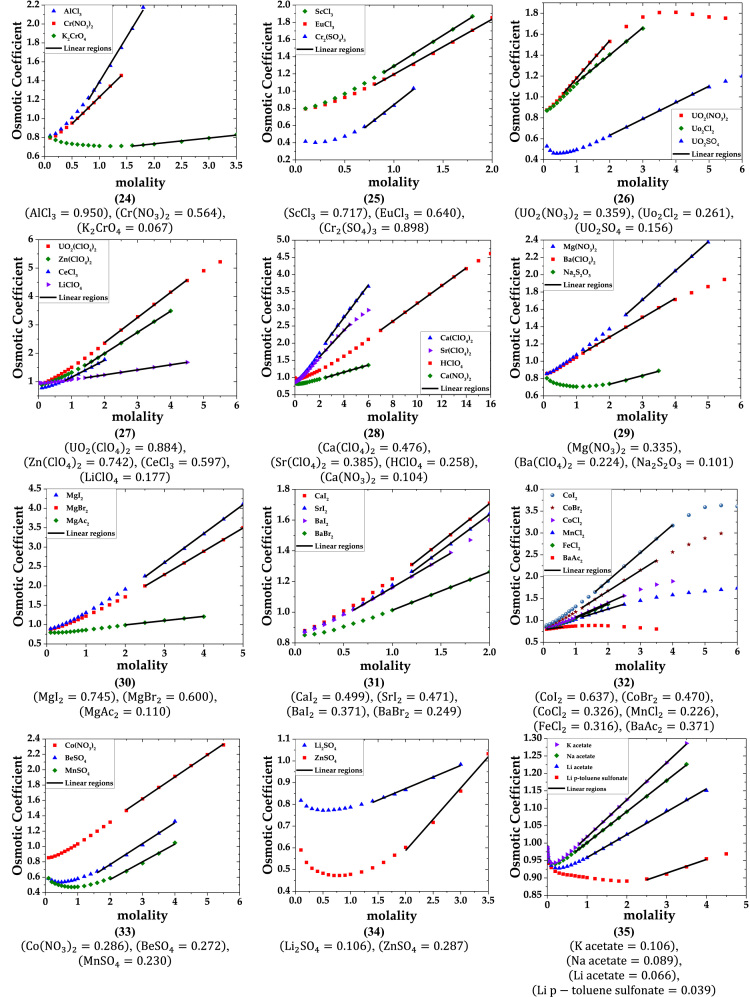

**Figure:**
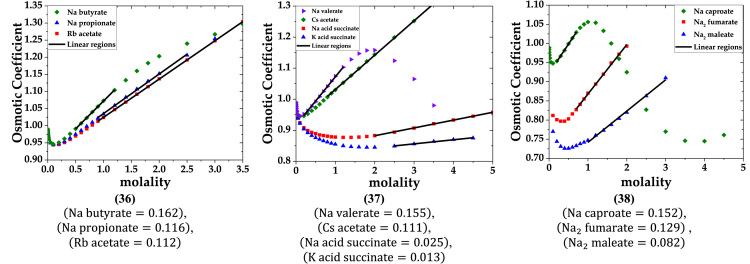

Part-2: Table of primary molar hydration volume and primary hydration number of electrolytes at 25 °C derived from the slopes of the linear regions of the plots presented in Part-1 (Table 1). The table also lists the molar volumes of bound water molecules.

**Table 1:** Primary molar hydration volume and primary hydration number of electrolytes and molar volume of bound water molecules at 25°C.

**Electrolyte**	**Primary molar hydration volume,**$v_{h}$$\times 10^{5}\;m^{3}\;{\mathrm{mol}}^{-1}$	**Molar volume of bound water,**$v_{w}^{b}$$\times 10^{5}m^{3}\;{\mathrm{mol}}^{-1}$	**Primary hydration number,**h
NaCl	7.975	1.496	4.2
LiCl	9.851	1.613	5.1
KCl	6.593	1.480	3.0
HCl	12.307	1.677	6.2
LiBr	11.247	1.608	5.8
KF	7.315	1.561	3.9
KI	7.869	1.501	3.0
CsCl	7.382	1.490	3.1
HNO_3_	9.083	1.551	4.2
NaBr	9.361	1.583	4.6
NaClO_4_	7.917	1.502	3.2
RbCl	7.641	1.466	3.6
LiNO_3_	8.392	1.566	3.9
NaI	10.808	1.594	4.9
NaOH	9.398	1.606	5.3
MgCl_2_	14.581	1.561	7.3
CaCl_2_	17.0338	1.553	8.8
BaCl_2_	16.693	1.576	8.3
Mg(ClO_4_)_2_	23.693	1.653	10.8
Ca(ClO_4_)_2_	21.119	1.641	9.2
Ca(NO_3_)_2_	16.081	1.561	7.3
HCOONa	6.837	1.495	3.6
LiI	11.159	1.600	5.2
KOH	9.971	1.587	5.5
CsF	8.751	1.571	4.4
CsBr	7.760	1.469	3.2
NH_4_Cl	6.609	1.448	2.7
Na_2_SO_4_	12.559	1.521	3.9
(NH_4_)_2_SO_4_	13.069	1.399	5.2
CaBr_2_	16.014	1.629	7.4
Cd(NO_3_)_2_	10.125	1.571	3.5
CdBr_2_	7.515	1.446	2.5
CdCl_2_	6.189	1.372	2.1
CdI_2_	9.978	1.515	2.8
CdSO_4_	11.901	1.456	3.8
Co(NO_3_)_2_	13.346	1.609	5.5
CrCl_3_	15.647	1.635	6.7
MgSO_4_	10.214	1.417	2.8
Cu(NO_3_)_2_	18.036	1.597	8.5
CuCl_2_	13.342	1.569	6.5
LaCl_3_	25.317	1.626	12.5
Mg(NO_3_)_2_	17.0168	1.625	7.7
MnSO_4_	12.113	1.494	3.9
NdCl_3_	25.447	1.627	12.6
NiCl_2_	17.536	1.614	8.9
NiSO_4_	11.637	1.461	3.7
PrCl_3_	25.346	1.626	12.5
SmCl_3_	25.751	1.626	12.8
SrBr_2_	19.275	1.620	9.4
SrCl_2_	16.852	1.614	8.3
Zn(NO_3_)_2_	17.719	1.613	8.2
ZnCl_2_	11.826	1.573	5.5
ZnSO_4_	12.822	1.497	4.4

Comparison between the calculated primary hydration number for electrolytes and those reported in the literature using NMR and Extended X-ray Absorption Fine Structure (EXAFS) spectrometry has been present in our main paper [1].

## 2 Experimental design, material and methods

Slope s of the linear region of the plot is given by

(1) $$s={\left(\frac{c}{m}\right)}^{2}\frac{v_{h}^{2}}{{2M}_{w}\nu }$$

$v_{h}$ is the primary molar hydration volume of the electrolyte, $M_{w}$ is the molecular mass of water, ν is the number of ions produced on dissociation of one molecule of the electrolyte, m is the molality of the electrolyte solution, c is its molarity. Variation of c/m in the linear region is small and hence the average value of c/m is used. The values of $v_{h}$ at 25 °C are listed in the table of Part-2.

The primary hydration number h of an electrolyte is related to its primary hydration volume by the following relation

(2) $$v_{h}=v_{s}+hv_{w}^{b}$$

$v_{s}$ is the molar volume of the bare electrolyte and is calculated from the Pauling radii of the constituent ions using the formula $v_{s}=\frac{4}{3}\pi N_{\mathrm{av}}\sum_{i}r_{i}^{3}$. Where $N_{\mathrm{av}}$ is Avogadro number and $r_{i}$ is the Pauling radius of constituents ions. These radii are obtained from the references given in Table 2.

**Table 2:** References listing Pauling radii of ions.

**Ions**	**References to Pauling radius**
Li^+^, Na^+^, K^+^, NH_4_^+^, Rb^+^, Cs^+^, Mg^2+^, Ca^2+^, Sr^2+^, Ba^2+^, Zn^2+^, Cd^2+^, Mn^2+^, Ni^2+^, La^3+^, F^-^, Cl^-^, Br^-^, I^-^	[3] Robinson and Stokes
H_3_O^+^,Cr^3+^,Co^2+^, Cu^2+^, Nd^2+^, Pr^3+^, Sm^3+^,NO_3_^-^, SO_4_^2-^	[4] Marcus
OH^-^,	[5] Masterton et al.
ClO_4_^-^, HCOO^-^	[6] Roobottom et al.

The molar hydrated volume of electrolyte, $v_{h}$ is the summation of the molar hydrated volume of the cations, ${v_{h}}_{+}$ and the anions, ${v_{h}}_{-}$

(3) $$v_{h}=\nu_{+}{v_{h}}_{+}+\nu_{-}{v_{h}}_{-}$$

where $\nu_{+}$, $\nu_{-}$ are the number of cation and anion respectively upon dissociation of one molecule of the electrolyte.

where the molar hydrated volume of the cation, ${v_{h}}_{+}$ is given as follows

(4) $${v_{h}}_{+}={v_{s}}_{+}+h_{+}{v_{w}^{b}}_{+}$$

and molar hydrated volume of the anion, ${v_{h}}_{-}$ is given by the following expression

(5) $${v_{h}}_{-}={v_{s}}_{-}+h_{-}{v_{w}^{b}}_{-}$$

In Eqs. (4), (5), $h_{+}$ and $h_{-}$ are primary hydration number of cation and anion respectively.

$v_{w}^{b}$ in Eq. (1) is the molar volume of the water in the primary hydration shell (bound water). This volume is lesser than that of bulk water due to electrostriction caused by high electric field around the ion. The electrostatic field generated by the ion is estimated at the periphery of the hydrated ion using following equation

(6) $$E^{*}=\frac{q}{4\pi \varepsilon^{*}\varepsilon_{0}r_{h}^{2}}$$

where $r_{h}$ is the hydrated radius which is calculated using the formula $r_{h}={\left[v_{h}/\left(\frac{4\pi N_{\mathrm{av}}}{3}\right)\right]}^{1/3}$. $\varepsilon_{0}$ is the vacuum permittivity. $\varepsilon^{*}$ is the dielectric constant of bound water and is calculated using Booth Eq. [7]

(7) $$\varepsilon^{*}=n^{2}+\frac{\alpha \pi N_{0}\left(n^{2}+2\right)\mu_{v}}{E^{*}}L\left(\frac{\beta \mu_{v}\left(n^{2}+2\right)E^{*}}{\kappa T}\right)$$

where n is the optical refractive index, $N_{0}$ the number of molecules per unit volume, $\mu_{v}$ the dipole moment of the water molecule, $E^{*}$is the field strength, and T the absolute temperature. L(x) is the Langevin function, and α and β are numerical factors (α=4/3, β=1/2 as per Onsager model).

Solving Eqs. (6), (7) simultaneously, we can find values of both $E^{*}$ and $\varepsilon^{*}$. However, the second term of Eq. (7) is much smaller than $n^{2}$ and $\varepsilon^{*}=n^{2}$. At 25 °C, $n^{2}=1.769$. Therefore, the value of the dielectric constant of bound water is taken as $\varepsilon^{*}=1.769$ for all calculations.

The pressure in the hydration shell is calculated using equation given by Desnoyers et al. [8].

(8) $$P^{*}\left(\mathrm{GPa}\right)=0.137+7.334\times 10^{-3}\left(E^{*}\left(\mathrm{GV}\;m^{-1}\right)\right)+4.599\times 10^{-3}{\left(E^{*}\left(\mathrm{GV}\;m^{-1}\right)\right)}^{2}$$

The values of $E^{*}$, obtained from the solution of Eq. (6) are used in Eq. (8) to obtain the pressure in the hydration shell. Then the density, $\rho_{b}$ of the bound water is obtained by the tables given by Grindley and Lind [9] which relates density of water to pressure. From the density, the molar volume of bound water is obtained using the equation $v_{w}^{b}=\frac{M_{w}}{\rho_{b}}$. The values of $v_{w}^{b}$ are listed in the second column of the Table 1. These values are used in Eq. (1) to calculate the primary hydration number which are listed in the last column of the Table 1.

We have provided the equation to be used for estimation of the excess nonelectrostatic contribution to the osmotic coefficient in our main paper [1] (See Eq. (8) of the main paper)

(9) $$\varphi_{e}^{\mathrm{NE}}=-\frac{1}{\nu mM_{w}}\left[\ln\left(1-cv_{h}\right)+c\left(v_{h}-\nu {\overset{®}{v}}_{w}\right)\right]-1$$

The nonelectrostatic contribution is given by

(10) $$\varphi^{\mathrm{NE}}=\;\varphi_{e}^{\mathrm{NE}}+1=-\frac{1}{\nu mM_{w}}\left[\ln\left(1-cv_{h}\right)+c\left(v_{h}-\nu {\overset{®}{v}}_{w}\right)\right]$$

Moreover, the Fig. 3 of our main paper shows the excess nonelectrostatic contribution to the osmotic coefficient for NaCl at 25 °C. It is seen that the excess nonelectrostatic contribution varies approximately linearly with the electrolyte concentration.
