# Supplementary material for: Data on primary hydration characteristics of aqueous electrolytes
Source: Data Brief. 2018 May 22;19:486–94. doi: 10.1016/j.dib.2018.05.037 (PMC5997881; doi:10.1016/j.dib.2018.05.037)
Supplement: Supplementary file 1 — Supplementary material [file mmc1.docx]

**Conflict of Interest Form**

There is no conflict.
